# Extraembryonic tissue in chelicerates: a review and outlook

**DOI:** 10.1098/rstb.2021.0269

**Published:** 2022-12-05

**Authors:** Nikola-Michael Prpic, Matthias Pechmann

**Affiliations:** ^1^ Justus-Liebig-Universitaet Giessen, Institut für Allgemeine Zoologie und Entwicklungsbiologie, AG Zoologie mit dem Schwerpunkt Molekulare Entwicklungsbiologie, Heinrich-Buff-Ring 38, 35392 Giessen, Germany; ^2^ Institute for Zoology, University of Cologne, Biocenter, Zuelpicher Strasse 47b, 50674 Cologne, Germany

**Keywords:** extraembryonic membranes, early development, Chelicerata, Arachnida, spider

## Abstract

The formation of extraembryonic membranes (EEMs) contributes to the proper development of many animals. In arthropods, the formation and function of EEMs have been studied best in insects. Regarding the development of extraembryonic tissue in chelicerates (spiders and relatives), most information is available for spiders (Araneae). Especially two populations of cells have been considered to represent EEMs in spiders. The first of these potential EEMs develops shortly after egg deposition, opposite to a radially symmetrical germ disc that forms in one hemisphere of the egg and encloses the yolk. The second tissue, which has been described as being extraembryonic is the so-called dorsal field, which is required to cover the dorsal part of the developing spider germ rudiment before proper dorsal closure. In this review, we summarize the current knowledge regarding the formation of potential extraembryonic structures in the Chelicerata. We describe the early embryogenesis of spiders and other chelicerates, with a special focus on the formation of the potential extraembryonic tissues.

This article is part of the theme issue ‘Extraembryonic tissues: exploring concepts, definitions and functions across the animal kingdom’.

## 1. Introduction

Tissues that form extraembryonic membranes (EEMs) are crucial for the correct embryonic development of vertebrate as well as many invertebrate organisms. EEMs are formed by cells of the fertilized egg, assist in the embryonic development and enable many organisms to deposit their eggs on land (e.g. [1]).

In many insects, the amnion and the serosa are the two EEMs that are laid down during early embryonic development. While the amnion covers the embryo ventrally, leading to a fluid-filled amniotic cavity, the serosa is close to the egg shell and covers the yolk, amnion and the developing embryo proper ([2], reviewed in [3]). Amnion and serosa are protective membranes. Apart from a simple mechanical protection against injury, extensive studies in the beetle *Tribolium castaneum* have shown that the serosa has an important function in protecting the egg against desiccation and providing innate immune response upon injury and infection [3–6]. While the desiccation resistance is owing to the secretion of a multi-layered cuticle, the Toll and immune deficiency (IMD) pathway get activated after bacterial infection [3,6,7]. Interestingly, in higher flies like *Drosophila melanogaster*, the serosa and amnion are strongly reduced and embryonic immune response is much lower compared to *T. castaneum* [8].

In different insect lineages, a gene duplication and diversification event of the homeotic selector gene *Hox3* has led to the establishment of several copies of the gene *zerknüllt* (*zen*) (e.g. *zen1* and *zen2* in the beetle *T. castaneum*). Several studies have revealed that *zen* is involved in regulating the specification and the morphogenesis of the insect serosa (e.g. [9–11]). These studies also showed that the *T. castaneum* EEMs are crucial for correct embryonic development, as they are involved in processes of dorsal closure [10,11]. While the amnion and serosa seem to be present in most insects, it has been claimed that EEMs homologous to amnion and serosa are absent from all other arthropods (crustaceans, myriapods and chelicerates) (summarized in [5]). Nevertheless, extraembryonic structures have been described for chelicerate groups like spiders, scorpions and pseudoscorpions (see below).

In this review, we summarize the current knowledge regarding the subdivision of chelicerate embryos (especially spider embryos) into embryonic and potentially extraembryonic tissues. We conclude that good evidence exists for the presence of genuine extraembryonic tissue in some chelicerate groups, but the currently available data are not yet sufficient to elaborate on possible homologies of these tissues to extraembryonic tissue in insects. On the other hand, the extraembryonic nature of some of the tissues in arachnids is difficult to decide, because the developmental processes have not yet been studied in sufficient detail.

## 2. Extraembryonic tissues in spiders (Araneae)

**Figure 1.**
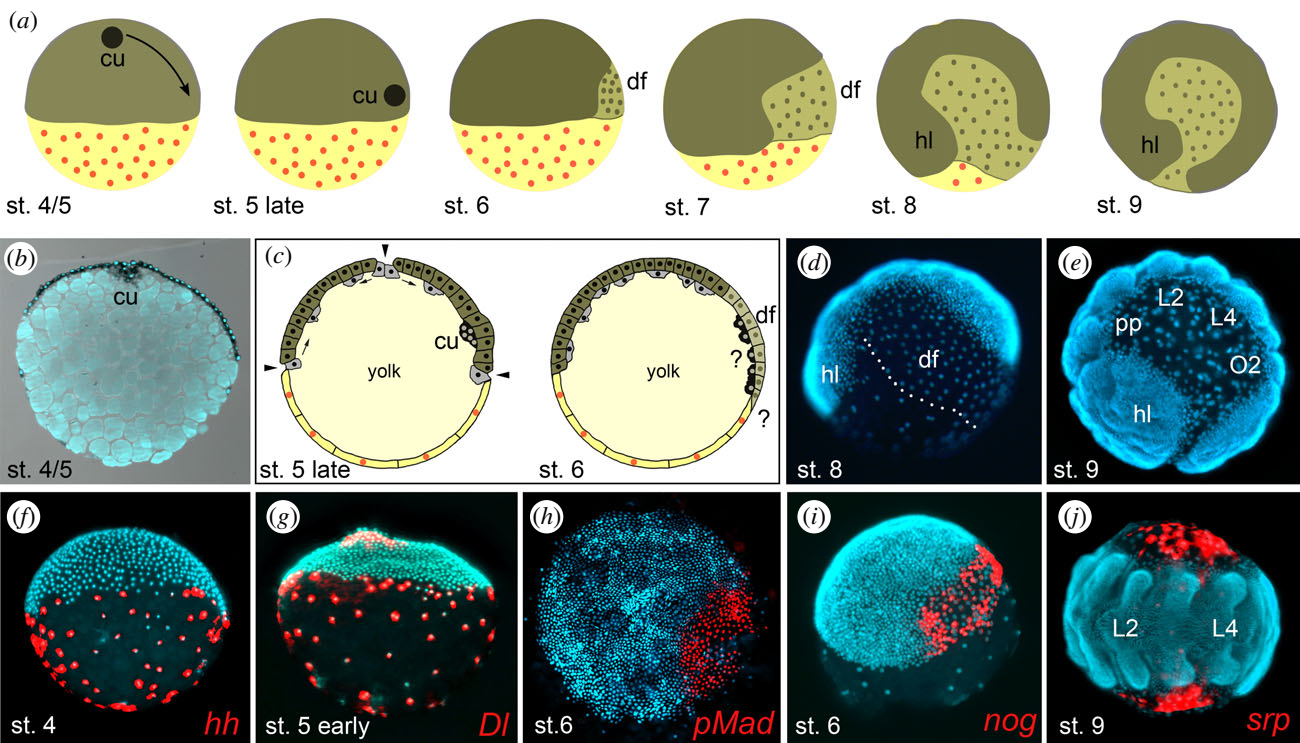
Overview of major developmental processes at germ disc and early germ band stages in the spider *Parasteatoda tepidariorum*. (*a*) Schematic drawings depicting externally visible development. At stage 4 and 5, the embryo comprises the germ disc (dark olive green) and the contra-orbital cells (yellow with the orange nuclei), both surrounding a central yolk mass. The cumulus (black dot) migrates (arrow at stage 4/5), approaches the disc rim at late stage 5 and induces the dorsal field (df; light olive green with olive green nuclei) at stage 6. Convergent extension mechanisms lead to the bilaterally symmetric germ band at stage 7. The dorsal field extends during further development (stage 8) until it covers the entire future dorsal side of the animal at stage 9. (*b*) Sagittal section of an embryo (stained for *armadillo* transcripts) at stage 4/5 to show internal features. The embryo is oriented such that the germ disc covers the upper half of the embryo. The germ disc is a single-layer epithelium. The yolk forms a solid inner mass and is divided into irregular yolk clods. The cumulus (cu) is a structure at the centre of the germ disc, comprising cells that intrude the yolk mass. Staining and sectioning of the embryo were performed as described in [19]. (*c*) Schematic cross-sections of embryos at late stage 5 and stage 6 to explain gastrulation processes. At late stage 5, cumulus cells (black) approach the rim of the germ disc (dark olive green) and are located between the central yolk mass (light yellow) and the germ disc. The contra-orbital cells are depicted in yellow with orange nuclei. In *P. tepidariorum* and other cobweb spiders like some *Latrodectus* species, it was demonstrated that at the former site of the cumulus, and at the rim of the germ disc, some cells (depicted in grey) ingress into the yolk (arrowheads), remain in close contact and then disperse across the internal surface of the germ disc epithelium (arrows) [20–22]. These internal cells have been identified as mesoderm and endoderm precursors [20–23]. The further fate of the cumulus cells after stage 6 is not clear (indicated by the question mark that is placed next to the black cumulus cells at stage 6). Live imaging and labelling of cumulus cells indicate that the cumulus cells disperse after cumulus migration and end up at several locations in the opisthosoma [16,20,24]. The dorsal field (light olive green) significantly expands after stage 6, but it is currently not known if the dorsal field cells spread over, intermingle or push back and replace the contra-orbital cells. This fact is indicated by the second question mark at the border between the dorsal field and the contra-orbital cells. (*d*) Embryo at stage 8, nuclei visualized by 4',6-diamidino-2-phenylindol (DAPI) counterstaining. The germ band corresponds to the area with a high density of nuclei; in the dorsal field, the nuclear density is lower, and nuclei of the contra-orbital cells are far apart from each other. Both dorsal field and contra-orbital cells are morphologically clearly separate from the germ band and therefore are usually regarded as extraembryonic tissue. (*e*) Embryo at stage 9, nuclei visualized as in (*d*). The germ band is still identified by the very crowded nuclei, clearly distinct from the lesser crowded nuclei in the dorsal field, but the contra-orbital cells with their widely separated nuclei are not visible anymore. (*f–j*) Patterning genes and signalling pathways are active in contra-orbital cells (*f,g*) and the dorsal field cells (*h–j*). At stages 4 and 5, the genes *hedgehog* (*hh*) (*f*) and *Delta* (*Dl*) (*g*) seem to be expressed in almost all contra-orbital cells. In the dorsal field cells at stage 6, bone morphogenetic protein (BMP) signalling is active, as indicated by the presence of phosphorylated Mad protein (pMad) (*h*), and the BMP antagonist *noggin* (*nog*) is also expressed in these cells (*i*). *Serpent* (*srp*) is expressed in lateral dorsal field cells at germ band stages ((*j*), ventral view). *In situ* hybridizations, DAPI counterstains, pMad antibody staining and false colour images of *in situ* hybridizations were produced as described in [19,25]. Staging after [26]. Additional abbreviations: hl, head lobe; pp, pedipalp; L, locomotory leg segment; O, opisthosomal segment.

The embryonic development of spiders has been studied in a large number of species from all major spider taxa (summary in [12,13]). Although taxon-specific features exist, the major processes of early embryonic development are the same in all araneids. The first cleavages affect only the cell nuclei, actual cell membranes are not present yet (e.g. [14], reviewed in [15]). These initial cleavages are thus reminiscent of the superficial cleavage in insects. The cleavage nuclei then move to the periphery of the egg, unite with portions of the cortical cytoplasm, and finally are encased with cell membranes to form proper blastomeres [14,16–18]. These cells thus form a thin epithelium, the blastoderm, that fully surrounds the central yolk mass. After blastoderm formation, a number of morphogenetic movements occur that may differ in divergent spider taxa, but the result of these movements is always the differentiation of the initially homogeneous blastoderm into two regions (figure 1): (i) a disc-shaped region comprising small cells that form a simple columnar epithelium, this region is generally termed the germ disc; and (ii) the remaining portion of the blastoderm comprising much larger and irregularly shaped cells. We term this region of the blastoderm the contra-orbital (opposite of the disc) region. Cell tracking and labelling in early embryos of the common house spider *Parasteatoda tepidariorum* have revealed that the majority of the blastodermal cells will contribute to the germ disc [27]. The contra-orbital region (comprising a squamous epithelium with large cells) is formed by a small portion of blastodermal cells that are directly opposite to the centre of the germ disc [27]. However, there are huge differences in the number of the contra-orbital cells, depending on spider species and egg size (e.g. [16,20,24,27,28]). Live imaging of embryos from remotely related spider groups (Araneomorphae and Mygalomorphae) has revealed that during gastrulation, cells do not invade the contra-orbital region. This observation strongly suggests that the strict separation into two hemispheres (germ disc and contra-orbital region) is conserved among all spiders [16,28].

During further development, the germ disc undergoes significant changes (overview in figure 1*a*). In the centre of the disc appears a round thickening, the cumulus. The cumulus forms by the invagination (or immigration) of cells that accumulate at the site of entry below the germ disc epithelium, in this way leading to the appearance of the visible thickening (e.g. [15,19,20,23,26–31]) (figure 1*b*). The cumulus then migrates from the centre in a straight line towards the rim of the germ disc. The significance of this migration of the cumulus that is observed in all spider species investigated so far is threefold: first, gastrulation at the original location of the cumulus internalizes additional cells that spread below the germ disc ectoderm during cumulus migration (figure 1*c*). These cells probably contribute to the mesoderm and parts of the endoderm that will form at later stages of embryonic development [19–23,29,32,33]. Second, the migration indicates the break of the radial symmetry of the germ disc and thus marks the beginning of the transition from the radially symmetric germ disc to the axially symmetric germ band with anterior–posterior and left-right polarity. Third, when the cumulus has reached the rim of the germ disc, it influences the local germ disc cells to change their morphology. The nature of this influence is currently unclear, but the result is, that the germ disc cells at the location where the cumulus has arrived become larger and change to a squamous cell shape. This area of the developing embryo is then called the dorsal field (reviewed in [15,29]).

Thus, at this stage, the spider embryo comprises three developmentally and morphologically separable areas: (i) the germ band, (ii) the dorsal field, and (iii) the contra-orbital region (figure 1*d*). Germ band and dorsal field both derive from the germ disc, but their further development differs quite substantially. The germ band undergoes segmentation and further morphogenesis towards the spider body plan (e.g. neurogenesis, appendage formation). It is therefore usually regarded as the ‘embryo proper’. By contrast, the dorsal field expands significantly during further development and represents the tissue that ‘closes’ the embryo on its future dorsal side, where the germ band is still open (before dorsal closure). The dorsal field is therefore similar in its location to e.g. the amnioserosa in *D. melanogaster*. Consequently, the dorsal field in spiders is widely regarded as extraembryonic tissue [15,21,29,31,32,34–39].

**Figure 2.**
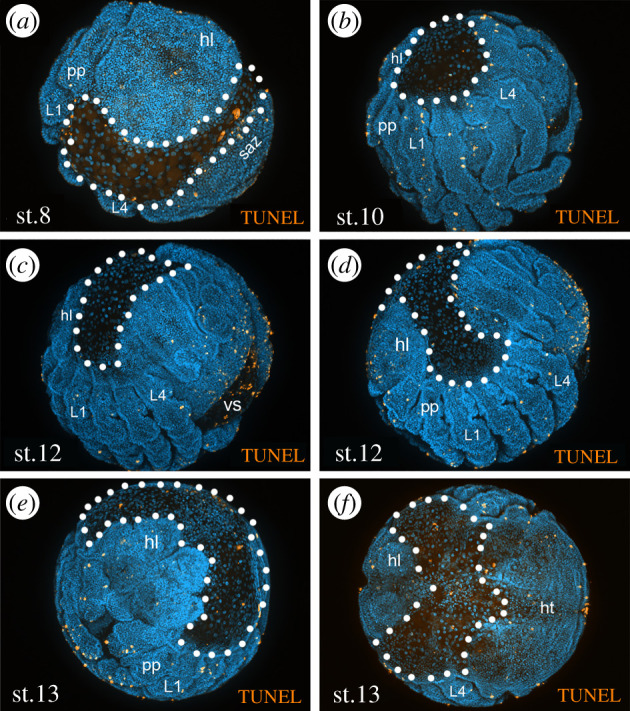
Analysis of cell death in *P. tepidariorum* via TUNEL staining. The dotted line indicates the area of the dorsal field, which was considered as extraembryonic tissue in previous studies. Only a few dying cells (in orange) can be detected in this area at stage 8 (*a*), stage 10 (*b*) and stage 12 (*c,d*). Somewhat elevated levels of cell death in the dorsal field are detected towards dorsal closure (stage 13, (*e*,*f*)). TUNEL detection of fragmented DNA in dead cells was performed using the In Situ Cell Death Detection Kit, TMR red (SIGMA-Aldrich) as described for *Drosophila* embryos (see [42]). Abbreviations: hl, head lobes; ht, heart tube; L1-L4, walking legs 1-4; pp, pedipalps; saz, segment addition zone; st, stage; vs, ventral sulcus.

Usually, the dorsal field is considered to be extraembryonic ectoderm [12], but Holm [24] presents evidence from tissue grafting and cell tracing experiments, that the dorsal field cells actually form an endodermal yolk sac, which is responsible for consuming the yolk and providing the developing embryo with nutrients [24]. The dorsal field therefore probably represents extraembryonic endoderm rather than ectoderm. The interpretation of the dorsal field as endodermal tissue is further supported by studies of the genes *serpent* (*srp*) and *hepatocyte nuclear factor 4* (*hnf-4*), which are strongly expressed in the dorsal field cells and later in the midgut (but also in other non-endodermal tissue) [34]. The fact that *srp* and *hnf-4* are expressed first in the dorsal field and later in the midgut could also indicate that (at least some) cells of the dorsal field later contribute to the midgut, but this is only speculative without further data on the fate of the dorsal field. It is difficult to draw any strong conclusions from the expression of single potential endodermal marker genes. For example, *srp* is expressed in a variety of cells in different insect species, even including the expression in extraembryonic cells like the amnioserosa of *D. melanogaster* or the cells of the amnion of *T. castaneum* embryos [40,41]. If the dorsal field is only a transient extraembryonic yolk sac that does not contribute to adult tissues and is destined to be removed towards the end of embryogenesis, one would expect it to show significant amounts of cell death. Unfortunately, the available data on cell death in the dorsal field are inconclusive. In *P. tepidariorum*, throughout germ band elongation, retraction and inversion stages, there is almost no cell death detectable in the dorsal field (figure 2*a–d*, [34]). Also in stages approaching dorsal closure, only a few more apoptotic cells can be detected in the dorsal field (figure 2*e,f*). However, a large number of dying cells were detected in the dorsal field of embryos of *Cupiennius salei* at comparable developmental stages [43]. Clearly, more work on cell death in the dorsal field and also data from more spider species is required to assess the role and significance of cell death in the dorsal field.

The developmental fate of the cells in the contra-orbital region is unclear. During the germ disc and early germ band stages, these very large cells take up more than half of the developing egg. As soon as the dorsal field starts developing and expanding, the contra-orbital cells become less and less visible, but it is unclear whether this is because they are pushed away, or eliminated or if they are simply overgrown by the advancing dorsal field (see question mark in figure 1*c*). When the dorsal field has reached its maximum expansion and has closed along the dorsal side, the contra-orbital cells are no longer visible (figure 1*e*). The presence of cells within the yolk mass, termed vitellophages (e.g. [12]), suggests that the vitellophages could be the remaining contra-orbital cells that have been overgrown by the dorsal field and have then entered the yolk mass. Unfortunately, the fate of the vitellophages is unknown. They received their name from similar cells in the yolk mass of insects, but if they actually have a role in the consumption of yolk (vitellophages = 'yolk eaters’) in spiders is entirely speculative. However, the contra-orbital cells are possibly also candidates for true extraembryonic cells in spiders, because if they are indeed required for yolk consumption and are then removed after the yolk has been used up, then they do not survive into post-embryonic stages.

## 3. Gene expression in dorsal field and contra-orbital cells in spiders

The molecular factors guiding EEM development in insects have been studied in several species. In the flies *D. melanogaster*, *Megaselia abdita* and *Episyrphus balteatus* and in the beetle *T. castaneum*, the derived Hox3 gene *zen* is required for the normal development of the EEMs (e.g. [9–11,44–46]). Specification of EEMs in the flies *M. abdita* and *D. melanogaster* additionally depends on signalling through the bone morphogenetic protein (BMP) pathway [47,48]. Further genes that are expressed in the insect EEMs include developmental genes like *Dorsocross* (*Doc*), *pebbled/hindsight, iroquois, pannier* and *tailup* (e.g. [49–52]). However, the evolutionary conservation of these factors in the establishment of putative extraembryonic tissues in chelicerates is unclear: the *zen*-homologue *Hox3* is not expressed in the dorsal field or the contra-orbital cells [53,54], and the expression and function of *Doc*, *hindsight*, *iroquois*, *pannier* and *tailup* have not yet been studied in chelicerates, whereas BMP signalling appears to be involved in the formation of the dorsal field in spiders (see below).

In spiders, a number of developmental genes have been identified that are expressed in the contra-orbital cells or the dorsal field. The genes *hedgehog* (*hh*) and *Delta* (*Dl*) are strongly expressed in all contra-orbital cells as of stage 3 of *P. tepidariorum* embryos (figure 1*f,g*), but their function in these cells has not yet been established. Throughout later embryonic development (stages 8–14), additional genes like *srp* (figure 1*j*) and *hnf-4* are strongly expressed in the dorsal field [34]. During stage 5, the shifting cumulus leads to the activation of the BMP signalling pathway in a subset of ectodermal germ disc cells that are located directly above the migrating cells of the cumulus (e.g. [19,29–31]). Several lines of evidence indicate that signals originating in the cumulus are crucial for the induction of the dorsal field. First, the transplantation of the cumulus is able to induce a secondary axis by establishing an additional dorsal field [16,24,37]. Second, laser ablation or removal of the cumulus or the knockdown of the BMP signalling pathway effectively block the establishment of the dorsal field [24,35,37]. Third, the ectopic activation of the BMP signalling pathway within the radially symmetric germ disc is sufficient to establish a secondary body axis by establishing an ectopic dorsal field [16]. Thus, germ disc cells in the area of influence of the signalling from the cumulus, activate BMP signalling (figure 1*h*) and develop into squamous dorsal field cells that gradually cover the yolk, left and right of the elongating germ band. Within the dorsal field, a few genes have been identified that likely depend upon BMP signalling for their activation or repression. BMP pathway activity is detectable during the complete process of lateral spreading of the dorsal field [35]. While the BMP antagonist *short-gastrulation (sog)* seems to be repressed in dorsal field cells, another BMP inhibitor *noggin* (figure 1*i*) and the pseudo-receptor *BMP activin membrane-bound inhibitor (bambi)* [55,56] show a strong expression within the cells of the dorsal field. Also, the transcription factors *forkhead* and *hunchback* are expressed in the dorsal field [30,38]. The persisting presence of BMP signalling in the cells of the dorsal field suggests that many of the BMP-regulated genes are not only required to establish the dorsal field, but are also required to complete the morphogenetic processes that lead to the spreading over the yolk.

It is interesting to note that BMP signalling is active in the EEMs of insects, where it is responsible to activate genes like *zen* and *Doc,* which are important factors for extraembryonic tissue maintenance and morphogenesis ([47,48], summarized in [51]). The squamous cell shape, the cellular behaviour to spread over large parts of the yolk or other cells of the egg and the strong activity of the BMP signalling pathway are strong similarities between the dorsal field of spiders and the serosa of insects. The proposed function of the dorsal field as an embryonic yolk sac based on cell labelling and tissue transplant experiments performed by Holm [24], and the expression of *srp* and *hnf-4* that both have previously been linked to endoderm development [34] suggests that the dorsal field is endodermal rather than ectodermal extraembryonic tissue.

## 4. Extraembryonic tissue in other arachnids

The Arachnida comprise 10 extant subgroups. Apart from spiders (Araneae), these are the scorpions (Scorpiones), pseudoscorpions (Pseudoscorpiones), whip scorpions (Uropygi), whip spiders (Amblypygi), palpigrades (Palpigradi), camel spiders (Solifugae or Solpugida), harvestmen (Opiliones), hooded tick spiders (Ricinulei) and the mites and ticks (Acari), the monophyly of which is, however, disputed [57]. For two of these subgroups, the Ricinulei and the Palpigradi, no information about their embryonic development is available yet, and the embryogenesis of the Solifugae is incompletely known (with a focus on the ultimate stages shortly before hatching) [58]. However, many general aspects of embryonic development as described above for spiders are very similar in other arachnids as well. Yolk-rich eggs are characteristic of arachnids in general, apparently leading to conserved mechanisms of early embryogenesis across arachnid taxa, although there are a few groups (e.g. certain scorpions and mites) with almost yolk-free eggs and different early developmental mechanisms (e.g. total cleavage). The formation of a germ disc and contra-orbital region is present in harvestmen [59,60], whip scorpions [61,62] and whip spiders [63,64], and also the later phases of development with the formation of the germ band and dorsal field are present in these arachnid groups. Based on the interpretation of the dorsal field in spiders as extraembryonic tissue, previous authors have regarded the dorsal field in these arachnid groups as extraembryonic tissue as well (summarized in [12]).

A somewhat different mode of embryonic development is seen in the Acari (mites and ticks). These do form a germ disc and contra-orbital region, and also a central thickening (cumulus) in the germ disc, but it is unclear whether the transformation of the germ disc into the germ band involves the migration of the cumulus to the rim of the disc [12,65,66]. Nevertheless, an extensive dorsal tissue similar in location and appearance to the dorsal field in spiders is visible in mite embryos as soon as the germ band has developed. In accordance with the assessment of the dorsal field as extraembryonic tissue, previous authors have regarded this tissue in Acari as extraembryonic tissue as well. The actual fate of this tissue in the Acari, however, is unclear.

**Figure 3.**
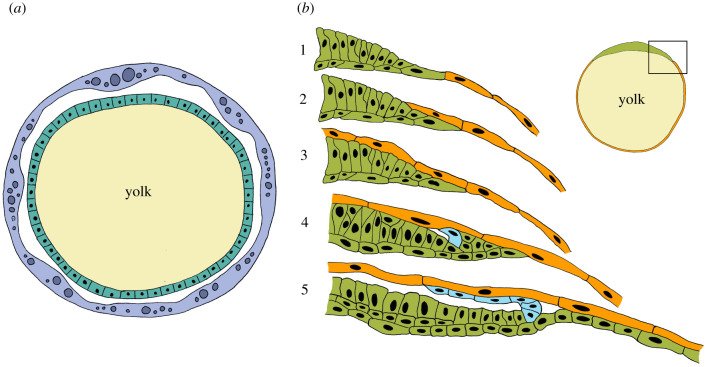
Uncontroversial EEMs exist in pseudoscorpions (*a*) and scorpions (*b*). (*a*) Diagram of a blastoderm stage embryo of a pseudoscorpion, simplified after Weygold [67]. The blastoderm (teal) is a simple epithelium surrounding a central yolk mass (beige). The entire blastoderm is surrounded by a thick EEM, the trophic membrane (lavender blue). Note the fluid-filled space between trophic membrane and blastoderm, which is believed to be crucial for normal gastrulation and further development [67]. (*b*) The formation of serosa and amnion in yolk-rich scorpion embryos according to Brauer [71]. At an early developmental stage, the blastoderm is differentiated into a denser area, or germinal disc (green) and the rest of the blastoderm with larger cells (orange) surrounding a central yolk mass (beige). The inset top right is an overview diagram, and a temporal sequence of development of the boxed area is depicted in panels 1 to 5. Initially, germinal disc cells and the larger blastodermal cells are continuous (1), but soon the larger blastodermal cells (orange) overgrow the germinal disc cells (green) (2 and 3). The entire orange epithelium is then called the serosa. After the serosa has formed, cells from the germinal disc begin to form an additional membrane, the amnion (light blue). Initially, single cells stand out by their change of shape (4), soon after these cells proliferate along the inner surface of the serosa (5) until they also fully cover the embryonic anlage (not shown).

Finally, scorpions and pseudoscorpions show some peculiarities that are not present in any of the other arachnid subgroups. In both groups, the embryos develop in close contact with the mother: in pseudoscorpions, the eggs are carried by the mother in a brood pouch on the opisthosoma and are supplied throughout embryogenesis with a nutrient fluid via the oviducts of the mother [67]. In scorpions, the embryos develop entirely within the body of the mother and the mother thus gives birth to fully developed nymphal instars. Scorpions are therefore regarded as viviparous or ovoviviparous [68–70]. The development of the embryos within the body or in a brood pouch of the mother, of course, requires the presence of specialized organs of the mother to supply the offspring with nutrients and other vital substances. However, the embryos of both groups show unique features that may be connected with their special mode of development within protective maternal organs. The early development of the pseudoscorpions deviates quite significantly from the early development of the other arachnid subgroups described above. Although the eggs are yolk-rich, the cleavages are total and result in a large number of small, yolk-free blastomeres (micromeres) and a few large blastomeres that contain the yolk (macromeres) [12,67]. The macromeres fuse to form a central yolk mass that is surrounded by the micromeres that form a thin blastoderm. Intriguingly, some of the micromeres do not contribute to the blastoderm, but move to the vitelline membrane, spread there across the inner surface of the vitelline membrane and fuse to form an additional layer of syncytial tissue that surrounds the entire embryo (figure 3*a*). This layer is termed the trophic membrane (or embryonic envelope), but its purpose is unclear. Weygoldt [67] has suggested that the primary function of the syncytial layer is to provide a fluid-filled space between the developing embryo and the vitelline membrane. Apart from this, it is generally assumed that the syncytial layer also serves as a storage device for nutritive fluid from the mother (hence the name trophic membrane). The fate of the trophic membrane is unclear. It becomes thinner and disappears at one point of development, but the mechanisms of its demise are unknown. Weygoldt [67] writes that the membrane is ‘eaten’ by the embryo, but this process has not been demonstrated. The further development of the pseudoscorpion embryo is also quite unique. There is no disc-shaped germ primordium, instead immigration or invagination of blastodermal cells occurs at several positions on the blastodermal sphere, leading to several visible thickenings [67]. One of these thickenings appears to be the blastopore, while other thickenings apparently represent the primordia of prosomal segments, the opisthosoma and the sucking organ (a unique embryonic organ in pseudoscorpions). Germ band formation thus does not seem to involve radial-axial symmetry transition, and there is also no obvious analogue of the dorsal field.

The development of scorpion embryos also shows a number of remarkable and unique features (summarized in [12,70]). Cleavage may be total or partial depending on the amount of yolk present in the egg. So-called katoikogenic species rely entirely on maternal supply and have little if any yolk at all, whereas the majority of species show the apoikogenic mode of development that at least partially relies on yolk supply. A blastoderm is formed that either surrounds a blastocoel or a yolk mass. The cells of the blastoderm on one egg pole are smaller and form the so-called germinal disc [71]. This germinal disc is ovoid (not circular like the germ disc in spiders) and thus already has axial symmetry and is equivalent to the germ band in other arachnids. The other end of the egg with less dense cells should then be regarded as the equivalent of the contra-orbital region. Local thickenings in the blastoderm have been described for scorpions, some of which have been called cumulus in the literature (e.g. [72]), but their relation to the cumulus in spiders and other arachnids is entirely unclear. No migration of such thickenings has been observed in scorpions, and similar to the situation in pseudoscorpions the germ band is apparently formed without any obvious transition from radial to axial symmetry. Likewise, no obvious counterpart of the dorsal field is present in scorpions.

Several putative EEMs have been described for scorpions and they have been compared to similar membranes in insects, and some of these membranes even have received the same names, serosa and amnion (summarized in [5]). Embryos of katoikogenic scorpions are surrounded by three different membranes: embryonic capsule, trophamnion and second embryonic envelope (from inner to outer). All of these membranes either disappear during development or are shed before birth. Their developmental origin, however, is unclear. Francke writes that the embryonic capsule and the second embryonic envelope derive from follicle cells [73]. If this is correct, then this is tissue of the mother, not of the offspring. Francke also states that the trophamnion derives from polar body cells, which would be remarkable, but would argue for the trophamnion being a genuine EEM [73].

By contrast, apoikogenic scorpion embryos are surrounded by only two membranes: amnion and serosa. The names suggest homology to the insect EEMs or seem to imply similar developmental formation or function. However, the development and the function of the scorpion amnion and serosa are not well understood, and the published accounts are inconclusive. Francke writes that the serosa is formed first and derives from the non-germ band portion of the blastoderm, whereas the amnion is formed only later in development and derives from cells of the germ band (figure 3*b*) [73]. This is in agreement with earlier studies by Metschnikoff, Laurie and Brauer [71,74,75]. By contrast, Abdel-Wahab [76] describes the simultaneous formation of serosa and amnion via an amnioserosal fold originating directly in front of the anterior rim of the germ band. The amnioserosal fold then supposedly stretches towards the posterior end of the germ band and fuses with the blastoderm there. In this way, the inner epithelium of the amnioserosal fold becomes the amnion, and the outer epithelium of the amnioserosal fold becomes the serosa. In other accounts of serosa formation, the non-germ band portion of the blastoderm detaches from the germ band and then grows around the germ band [12].

## 5. Extraembryonic tissue in non-arachnid chelicerates

Apart from the Arachnida, the Chelicerata comprise only two further extant subgroups, the horseshoe crabs (Xiphosura) and the sea spiders (Pantopoda or Pycnogonida). Both groups are marine and develop via typical larval stages, the trilobite larva in Xiphosura and the protonymphon larva in Pantopoda. Thus, the primary goal of embryogenesis in these groups is not the development of the adult body plan, but the formation of the larval body plan. The adult body plan is formed later from the larval body plan via metamorphosis. The early embryonic development of both groups deviates from the developmental modes in arachnids and often involves the subdivision of the blastomeres into large macromeres and small micromeres (e.g. [12,72,77]). The micromeres appear to be the embryo proper, but the fate of the macromeres is unclear, thus making any statement about their nature as potentially extraembryonic tissue difficult.

In sea spider species with eggs containing little or no yolk, early cleavage is total and equal and leads to a solid blastula (no blastocoel is present), followed by gastrulation processes that combine further cleavage, cell immigration and epibolic movements [72,78]. Thus, no proper germ band is formed and a separation into a germ disc/germ band and contra-orbital region never occurs in these species. In sea spider species with a higher amount of yolk in the eggs, the cleavage is less well understood. There is evidence that early cleavage is total, but unequal and produces micromeres and a smaller number of larger macromeres. Gastrulation is then described as an epibolic movement of the micromeres over the macromeres [77]. However, accounts of sea spider embryos with yolky eggs figure incomplete cleavage (at least partially), and the later subdivision of the entire embryo into a disc-shaped blastoderm (from which the primordium of the protonymphon larva develops) and a yolk mass that includes cell nuclei, but no or incomplete cell membranes [72,77].

In horseshoe crabs, the eggs are very yolk-rich [79]. The mode of cleavage of this huge amount of yolk is unclear in the literature. Most accounts state that cleavage is total [12,80], despite the yolk mass, whereas other authors regard the cleavage as incomplete or superficial (e.g. [72,81]). Regardless of the exact mode of early cleavage, the result is a blastoderm comprising irregularly shaped blastomeres, larger ones at one pole, smaller ones at the opposite pole. The region comprising the smaller blastomeres forms a germinal disc, and at the centre of the disc, an invagination indicates the process of gastrulation. The fate of the larger cells in the opposite half of the egg is unclear. The yolk also contains a large number of cell nuclei, but their origin and fate are unclear. They are usually regarded as vitellophages, i.e. loose extraembryonic cells that are thought to be involved in nutrient uptake from the yolk, but degenerate once the yolk is used up. However, Kimble *et al*. [82] have proposed that the cell nuclei in the yolk become incorporated into cells later in development and apparently become the midgut primordium in the trilobite larva.

## 6. Conclusion

A prime candidate for an evolutionarily conserved EEM in chelicerates is the dorsal field, which is present in Araneae, Opiliones, Amblypygi and Uropygi. In addition, the extensive dorsal tissue in Acari might correspond to the dorsal field of the other groups, but its origin (without the influence of the cumulus?) and its fate are unclear. The dorsal field is morphologically separated from the germ band proper, and it does not seem to contribute to the actual body of the animal, but instead it merely ‘covers the open dorsal side’ of the developing germ band. In fact, the location and apparent fate of the dorsal field is strikingly similar to the amnioserosa of higher flies such as *D. melanogaster*: both tissues stretch towards dorsal, starting from the rim of the germ band, both tissues cover the ‘open’ dorsal side of the animal before dorsal closure and both tissues are apparently gone when dorsal closure is complete. If not homology, this at least suggests the analogy of dorsal field and amnioserosa, and indeed the dorsal field is usually labelled as ‘extraembryonic ectoderm’ in the literature. It is surprising therefore, that data on cell lineage, tissue fate and gene expression of *srp* and *hnf-4* instead point to an endodermal nature of the dorsal field.

By using single-cell RNA sequencing, recent studies independently identified genes that are expressed in the dorsal field (e.g. *noggin*, see figure 1*i*; Akiyama-Oda *et al*. [83]; Leite *et al*. [84]). First analyses of cell clusters and the genes expressed therein led to the suggestion that the cells of the dorsal field are indeed not extraembryonic but might contribute to the formation of hemocytes and other cell types [83]. The analysis of these novel marker genes will enable us to better understand extraembryonic membrane formation and patterning and will help to better distinguish between extraembryonic and embryonic cells.

Are there other structures in arachnids (or chelicerates as a whole) that can serve as good candidates for extraembryonic tissues and membranes? The contra-orbital region that is present in Araneae, Opiliones, Amblypygi, Uropygi and Acari is another prime candidate for genuine extraembryonic tissue in chelicerates. By definition, these are the cells that are excluded from the germ disc, and thus are excluded from the tissue that will form the embryo proper. Although the actual fate of the contra-orbital cells is unclear, the available data are consistent with the notion that the contra-orbital cells represent extraembryonic tissue, the main purpose of which is simply to ‘close’ the blastoderm around the massive yolk content in these eggs. Another function of these cells might be to guide the cells of the dorsal field during their dorsal expansion. After the dorsal field has fused along the dorsal side and has taken over the role of the ‘dorsal’ tissue before the proper dorsal closure, the contra-orbital cells are no longer required and may be removed. Clearly, research into the origin, development and fate of the contra-orbital cells in arachnids, using the embryological tools and techniques available today, is very much needed, before we can even begin to speculate about the possible homology of the contra-orbital cells in arachnids to extraembryonic tissues in other arthropods.

The Xiphosura and the Pantopoda neither show a dorsal field nor is there a clear-cut equivalent of the contra-orbital cells. In xiphosurans and in pantopod species with yolk-rich eggs, there are cells opposite of a disc-shaped embryonic primordium. However, the fate of these cells is unclear, they might be extraembryonic cells, but they as well might contribute to embryonic tissues like midgut epithelium or muscles; their nature thus cannot be decided without further research on their developmental contribution and fate. The cells in the yolk mass of xiphosurans might be extraembryonic in the sense that they do not contribute to the larval animal, but instead function as short-lived vitellophages; however, the high number of these cells is very unusual (usually arthropod yolk contains no or only few cell nuclei) and Kimble *et al*. [82] present evidence to suggest that these cells are actually precursors of the midgut primordium in the Xiphosura.

Uncontroversial EEMs exist in the Pseudoscorpiones and Scorpiones. In pseudoscorpions, the young embryo is surrounded by a thick EEM, the trophic membrane. This membrane is a peculiarity of pseudoscorpions. It is undoubtedly not homologous to any other extraembryonic tissue in the arthropods and is therefore of little significance for our understanding of the phylogenetic interrelationships of EEMs in the phylum Arthropoda. Nevertheless, it is very unfortunate that the details of its origin and fate are unclear, because the separation of blastomeres from the forming blastoderm and their migration to the egg shell where they fuse into a syncytial inner lining of the vitelline membrane, are fascinating and unique developmental processes that can only be studied in pseudoscorpions.

In scorpion eggs with no or little yolk, the developing embryo is surrounded by three membranes, but for two of them, an origin from maternal tissue has been suggested. Only the trophamnion is believed to trace from the zygote and thus would qualify as extraembryonic tissue. However, its alleged derivation from polar body cells and its position between two maternally derived membranes, casts serious doubt on its extraembryonic nature; it is probably of maternal derivation as well. In scorpions with yolky eggs, the germ band is surrounded by two membranes of undoubtedly extraembryonic tissue. These membranes resemble EEMs in insects so closely, that they have even received the same names: serosa and amnion. However, the origin and development of the scorpion membranes is unclear. Therefore, in fact, no comparison with the origin and development of the insect membranes can be made yet. In addition, the insect amnion and serosa are required to protect the developing embryo against desiccation [5], whereas the scorpion membranes are unlikely to have a similar function, because the embryos develop within the body of the mother and therefore are not at risk of desiccation.

Given the recent trends in chelicerate phylogeny reconstruction [85], it is also interesting to ask whether the investigation of extraembryonic tissue in chelicerates can contribute to this discussion. The early development of the Xiphosura with its apparent lack of contra-orbital cells and dorsal field does not seem to support the new placement of the Xiphosura within the Arachnida [85]. The possession of well-developed EEMs in Pseudoscorpiones and Scorpiones, on the other hand, could be taken as a character supporting the placement of both taxa in a monophyletic group that has been termed Panscorpiones [85]. However, our review of the current status of the knowledge about the origin and fate of extraembryonic tissue in Chelicerata shows that many aspects of these processes are virtually unknown. Xiphosuran early development, for example, has not been investigated in sufficient detail as to rule out the presence of cell populations homologous to contra-orbital cells and dorsal field cells. Also, we know too little about the molecular, cytological and genetic mechanisms of the development of the EEMs in Pseudoscorpions and Scorpions to even ponder homologies between these membranes. Therefore, further research on the comparative developmental biology of EEMs in chelicerates is very much needed.

## Data Availability

This article has no additional data.
